# Multi-Method Assessment of Sleep in Children With Angelman Syndrome: A Case–Controlled Study

**DOI:** 10.3389/fpsyt.2019.00874

**Published:** 2019-11-29

**Authors:** Jayne Trickett, Chris Oliver, Mary Heald, Hayley Denyer, Andrew Surtees, Emma Clarkson, Paul Gringras, Caroline Richards

**Affiliations:** ^1^Cerebra Centre for Neurodevelopmental Disorders, School of Psychology, University of Birmingham, Birmingham, United Kingdom; ^2^Department of Health Sciences, College of Life Sciences, University of Leicester, Leicester, United Kingdom; ^3^Forward Thinking Birmingham, Birmingham Women’s and Children’s NHS Foundation Trust, Birmingham, United Kingdom; ^4^Great Ormond Street Institute of Child Health, University College London, London, United Kingdom; ^5^The Huntercombe Group, Worcestershire, United Kingdom; ^6^Evelina London Children’s Sleep Medicine Department Guy’s and St Thomas’ NHS Foundation Trust, London, United Kingdom

**Keywords:** sleep, actigraphy, Angelman syndrome, intellectual disability, case–control

## Abstract

**Objectives:** To assess sleep quality and timing in children with Angelman syndrome (AS) with sleep problems using questionnaires and actigraphy and contrast sleep parameters to those of typically developing (TD) children matched for age and sex.

**Methods:** Week-long actigraphy assessments were undertaken with children with AS (n = 20) with parent-reported sleep difficulties and compared with age and sex matched TD controls. The presence of severe sleep problems was assessed using the modified Simonds and Parraga sleep questionnaire. Sleep hygiene was measured using the Family Inventory of Sleep Habits.

**Results:** Actigraphy and parent-completed sleep diary data indicated that children with AS had significantly earlier bedtimes (p = .003, Cohen d = .47) and poorer sleep efficiency (78%, p = .04, d = .33) than TD children (84%). No significant differences in total sleep time, sleep onset latency or wake after sleep onset were found between the two groups. The expected relationship between later bedtimes and increasing age found for the TD group (p < .001, β.78) was not evidenced for the AS group (p = .09, β.39). Considerable inter-individual and night to night variation in actigraphy assessed total sleep time and wake after sleep onset was found for children with AS compared to TD children. Parent report indicated that a greater proportion of children with AS had severe night waking problems compared to TD children (81 versus 5%). No significant differences in sleep hygiene and excessive daytime sleepiness were found between the two groups (p > .05).

**Conclusions:** This study reports the largest objective dataset of sleep quality parameters in children with AS. Sleep quality in this group was characterised by poor efficiency and significant intra- and inter-individual variability that warrants further investigation. This variability should inform assessment and intervention for sleep in children with AS, as averages of total sleep, even across a 7 day period may not capture the difficulties with night waking highlighted by parental questionnaire report.

## Introduction

Angelman syndrome (AS) is caused by lack of expression of the *UBE3A* gene on the maternal 15q11–13 chromosome, arising from various genetic mechanisms (1). These mechanisms include a 5–7 Mb *De Novo* interstitial deletion of the maternal 15q11.2–Q13 region for approximately 75% of individuals, Paternal Uniparental Disomy (UPD) (1–2%), an Imprinting Defect (1–3%), and mutation in the *UBE3A* gene (10%) (2).

Several physical and behavioural features are consistently associated with AS, which include severe developmental delay, ataxic gait, frequent smiling/laughing, repetitive arm movements and limited or absent speech (3). Other characteristics present in over 80% of individuals include microcephaly, seizures, and abnormal EEG (3). Sleep disturbances, including insomnia and general sleep disorders or difficulties, are widely reported in AS (4). A systematic review of studies investigating sleep disturbances in children with genetic syndromes reports prevalence rates of sleep disturbances in children with AS of between 48% and 70% (5). Sleep disturbance in AS may occur downstream from impaired *UBE3A* expression resulting in the dysfunction of GABA receptors (6, 7). A number of studies have suggested that individuals with AS do experience circadian rhythm disorders (8) evidenced by a shift in melatonin offset timings relative to TD children and children with epilepsy (9). However, whether individuals with AS secrete a smaller volume of endogenous melatonin than peers is unclear (8, 9). More specifically, Takaesu et al. (8) identified 53% of their sample of 15 individuals with AS to have a circadian rhythm disorder, including an irregular sleep–wake cycle disorder (26.7%), non-24-h sleep–wake cycle (13.3%), and delayed sleep phase disorder (13.3%). Impaired circadian rhythm implicating the activity of E6-AP encoded by the affected *UBE3A* gene specific to AS has been hypothesized (10). Thus, in the investigation of sleep disturbance in AS, there is a need to characterize the specific sleep disturbances experienced by children with AS using validated questionnaires and objective measures and evaluate sleep phase, given possible circadian rhythm disorders.

Specific problems relating to sleep reported in children with AS include increased sleep onset latency, bedtime resistance, anxiety, rhythmic movements whilst falling asleep, nocturnal wakings, sleep disordered breathing and daytime sleepiness (6). Prevalence rates for night waking range from 60 to 100%, with a decrease in waking observed with increasing age (11). Variability in reported prevalence rates may be due to different definitions of night waking used across studies. When quantifying the severity of night waking as three or more times a week and lasting over several minutes, the prevalence was 46% of children with AS (mean age 8.64 years) (12).

Little is known about the impact of sleep disturbance on children with AS. Daytime sleepiness according to single items on questionnaires is reported for only between 8 and 24% of children (6, 13–15) and represents a small effect size (.10) in a meta-analysis (16). This raises questions as to whether children with AS have a reduced need for sleep and whether shortened total sleep time negatively impacts upon children’s daytime functioning. Whilst the behavioural impact of shortened sleep duration has not been studied in children with AS, children with severe intellectual disabilities with sleep problems have a greater severity of challenging behaviour (17). The reduced total sleep time may be due to increased sleep onset latency, reported for between 32% and 91% of individuals (6, 13, 18), in combination with reduced sleep efficiency due to night wakings (7, 19). The range of definitions for night waking and the inclusion of young adults in some of these studies limit the conclusions that can be drawn from these data. Therefore, there is a need to identify how poor sleep in AS is characterised using objectively assessed total sleep time, sleep onset latency, and duration of night waking using a clearly defined age range of children with a parent-reported sleep problem compared to a control group of TD children.

Few studies have used objective measures to provide detailed descriptions of sleep quality in children with AS; one used polysomnography (20) and two studies employed actigraphy with samples of ≤13 children (19, 21). Actigraphy assessment over a minimum of 7 days for 10 children with AS aged 2 to 16 years, showed means for children’s total sleep time of 406 min (SD 55), wake after sleep onset 114 min (SD 33), and sleep onset latency of 41 min (SD 23) (19). The authors commented upon the extended night waking; however it is important to compare actigraphy assessed night waking to that of TD children, in order to draw conclusions about the severity of impaired sleep in children with AS. A larger actigraphy study of children with AS is needed to overcome the limitations of the existing literature.

As sleep in childhood is sensitive to developmental maturation, it is also important to assess sleep quality and timing in atypical populations compared to an age-matched sample of TD children which is lacking in previous studies. The advantage of actigraphy assessment of sleep is that it is unobtrusive and sleep timing and quality can be monitored across multiple nights, unlike one night polysomnography. This would enable night to night variation, hereafter referred to as intra-individual variation, in sleep quality to be assessed. The assessment of this sleep parameter is important, as greater intra-individual variation in sleep offset timing is associated with more externalising, inattentive and aggressive behaviour in TD pre-school children (22). Aggression is frequently reported in individuals with AS (73% prevalence) (23), therefore identifying correlates of challenging behaviour in this group would elucidate the hypothesis that poor long-term sleep quality due to sleep variability does impact upon daytime functioning in children with AS.

In addition to the comparison of sleep quality and timing of sleep in children with AS who have a parent reported sleep disturbance compared to that of age-matched TD children, the cross-sectional associations with age in children with AS need to be compared against those of TD children. TD children in this study provide a benchmark for typical sleep quality for a child without AS of the same age. Currently, the profile of sleep problems across the developmental trajectory of AS is unclear. Retrospective caregiver report from adolescents and adults with AS indicate that sleep quality and daytime sleepiness improved for 65% of the sample (24). In adulthood, only 56% of a sample of 53 individuals slept for 8 h or more according to caregiver report (25). Other cross-sectional studies using age as a correlate or comparing between arbitrary age groups have suggested that sleep difficulties persist with age (15, 6). However, these studies did not compare age associations with sleep outcomes relative to an age-matched control group. Actigraphy assessments paired with sleep diaries would enable sleep timing to be correlated with age.

The aims of this study are to: 1) describe the profile and variability of sleep quality and quantity in children with AS with a reported sleep problem across a week long assessment, 2) compare symptoms of sleep disturbance, specifically sleep disordered breathing, excessive daytime sleepiness and the role of sleep hygiene in children with AS, to those shown by TD children, and 3) determine the association between age and total sleep time and bedtime for each group and compare across groups.

## Materials and Methods

### Participants

Twenty-two children with AS with a parent reported sleep disturbance were recruited from Angelman UK (Angelman syndrome UK support group) and from an existing database of families at the Cerebra Centre for Neurodevelopmental Disorders. Whilst no statistically significant differences in sleep disturbance severity between the genotypes of AS are reported (26, 27, 6) there are differences in rates of seizures and motor impairments between the AS genotypes (28). Therefore, to cater for known differences between the groups, only children with the UBE3A deletion subtype of AS were included. Two children did not tolerate the Actiwatch for the required four nights and their data were excluded from the study. Typically developing (TD) children were age-matched to each child with AS and were recruited through social media and through family and friends of staff at the Cerebra Centre for Neurodevelopmental Disorders. The mean age of children with AS was 9.4 years (SD 3.7) (see **Table 1**). Neither age, nor sex differed significantly between the groups. No difference in maternal education (*X^2^* = 2.42, p = .658) or family income was found between the two groups (*X^2^* = 6.78, p = .342). Medications used by children with AS are presented in **Table S1**.

**Table 1:** Demographic characteristics.

		AS	TD	T/X^2^	P value
Age mean (SD)		9.43 (3.72)	9.62 (3.71)	T = .155,	.879
Males n (%)		8 (40.0)	9 (45.0)	X^2^ = .102,	.749
Adaptive Behavior Composite score VABS standard score mean (SD)		43.95 (9.17)	–	–	–
Able to walk unaided n (%)^a^		7 (37)	20 (100)	18.25	<.001
Ever experienced tonic–clonic seizures		9 (45)	–	–	–
Ever experienced absence seizures		20 (100)	–	–	–
Ever experienced clonic seizures		4 (20)	–	–	–
Ever experienced myoclonic seizures		7 (35)	–	–	–
Ever experienced tonic seizures		2 (10)	–	–	–
Ever experienced atonic seizures		9 (45)	–	–	–
Ever experienced focal seizures		6 (30)	–	–	–
Ever experienced unknown classification of seizures (e.g. epileptic spasms)		3 (15)	–	–	–
Medically refractory epilepsy^b^		3 (20)	–	–	–
Using medication to aid sleep n (%)^c^		13 (65.0)	–	–	–
Medication helpful to aid sleep^d^		10 (76.9)	–	–	–
Maternal education^e^	Fewer than 5 GCSE’s or O Level’s (grades A–C), NVQ 1 or, BTEC First Diploma	2 (10.0)	0	X^2^ = 2.42	.658
	5 or more GCSE’s or O Level’s (grades A–C), NVQ 2, or equivalent	2 (10.0)	2 (10.5)		
	3 or more “A” Levels, NVQ 3, BTEC National, or equivalent	1 (5.0)	1 (5.3)		
	Polytechnic/university degree, NVQ 4, or equivalent	11 (55.0)	10 (52.6)		
	Masters/doctoral degree, NVQ 5, or equivalent	4 (20.0)	6 (31.6)		
Family income^f^	Less than £15,000	2 (10.5)	0	6.78	.342
	£15,001 to £25,000	2 (10.5)	4 (21.1)		
	£25,001–£35,000	2 (10.5)	1(5.3)		
	£35,001–£45,000	4 (21.1)	2 (10.5)		
	£45,001–£55,000	2 (10.5)	6 (31.6)		
	£55,001–£65,000	3 (15.8)	1 (5.3)		
	£65,001 or more	4 (21.1)	5 (26.3)		

a One missing response AS group.

b Defined as defined as inadequate seizure control despite appropriate medical therapy with at least 2 Anti-epilepsy drugs in maximally tolerated doses for 18 months–2 years, or adequate seizure control with unacceptable drug-related side effects (29) 5 missing responses.

c One parent indicated that clobazam administered in the evening was helpful to aid sleep. Included this response in using medication to aid sleep. Clobazam was administered to three other children in the absence of other medication to aid sleep. As no comment of its efficacy for sleep was reported, these three children were excluded from the total.

d One missing response AS group.

e One missing response TD group.

f One missing response AS group, 1 missing response TD group.

### Procedure

Consent was obtained from parents of participants. The study received favourable ethical review from the University of Birmingham. Families were instructed that their child should wear an actigraph for seven nights of continuous wear where possible. For children with AS a researcher visited the family to set up video equipment to record night-time sleep behaviours (see 30) and instruct on sleep diary completion and operation of the event marker on the actigraph. A comparable training video was sent to parents of children in the TD group. Parents completed a questionnaire pack and this was returned by post or the pack was collected in person by the researcher. The Vineland Adaptive Behavior Scales-2 (31) was completed either over the phone or in person with the family of children with AS. The measures and recruitment of TD children in this study replicate those used in study of sleep in children with Smith–Magenis syndrome (32).

### Measures

A questionnaire was completed by parents to collect information about children’s medication use, epilepsy, maternal education and family income. Adaptive ability in the AS group was assessed using the Vineland Adaptive Behavior Scales-2 Interview. A standardised behaviour composite score for the sample was reported. No measure of ability was used in the TD group; chronological age was assumed to be commensurate with developmental age as no statements of additional learning needs were indicated.

#### Symptoms of Sleep Disturbance and Sleep Hygiene Questionnaires

Severe sleep disturbance was assessed using the Modified Simonds and Parraga sleep questionnaire (33, 34) which has been validated for use with individuals aged 2 to 16 with an autism spectrum disorder (34). The presence of severe night waking, settling problems and early morning wakings were derived from this questionnaire based on the frequency (many times a week or daily) and intensity of the problems (e.g. night waking—takes over a few minutes to fall back to sleep; settling—takes over an hour to fall asleep).

The sleep-related breathing disorders screening questionnaire is a 22-item informant report measure from the Pediatric Sleep Questionnaire used to assess risk for sleep-related breathing disorders, which includes children with obstructive sleep apnoea and upper airway resistance syndrome (35). The 22 items relate to sleepiness, behaviour, snoring, and breathing subscales and sleep-related breathing disorder subscale. Items from the snoring subscale only were reported as other subscales require the child to be able to communicate their internal state.

Excessive daytime sleepiness was assessed using the modified Epworth Sleepiness Scale (MESS; 35). Parents/carers rate the likelihood of their child falling asleep in eight different situations (0 to 3), with higher scores indicating a greater likelihood. The questionnaire is based on the Epworth Sleepiness Scale (ESS) for adults. The ESS has good reliability and validity (36). The MESS has been used previously with children with intellectual disabilities and neurodevelopmental disorders (37). As the majority of children with AS are nonverbal, the question referring to ‘sitting and talking to someone’ was modified by the authors to include ‘sitting and talking to or interacting with someone.’ Seven out of the eight questions used in the MESS in the present study are similar to those used in a validation study of the ESS for Children and Adolescents (38) with individuals aged 12–18 years, which showed strong test-retest reliability and high internal reliability (39). In the present study a cut off score of >10 was used to identify children at risk of excessive daytime sleepiness.

Sleep hygiene was assessed using the Family Inventory of Sleep Habits (FISH; 40) developed from a sample of children with autism spectrum disorder. Items are scored on a five-point Likert scale, with higher scores indicating better sleep habits. The test-retest reliability of the measure with children with autism spectrum disorder is .83, and in the TD population is .59. The FISH also has good external validity with measures of childhood sleep (40).

#### Actigraphy

Sleep quality was assessed using the Actiwatch 2, manufactured by Philips Respironics. This accelerometer’s sampling rate is 32 Hz and 30 s epochs were used. Sleep onset and offset were defined as the clock times at the start of the first of 10 min scored as sleep (after lights out time) and the end of the last 10 min scored as sleep respectively. Wake After Sleep Onset (WASO) was detected according to the device’s medium sensitivity (40 counts per epoch). All other parameters were calculated according to default Actiware version 6.0.7’s settings, as these settings were found to have the greatest concordance with polysmonography (41). Compared to polysomnography, these settings have high sensitivity to detect sleep (.94) and specificity to detect waking (.69) in school-aged children (42)^1^. Parents were asked to press an event marker button at the child’s bedtime. Fifteen children with AS wore the Actiwatch on their ankle and five wore it on their wrist. Alternative actigraphy placement; a pocket in a cloth vest, has been used in another actigraphy study of children with AS (21). All TD children wore the Actiwatch on their wrist.

As an adjunct to actigraphy data, parents completed a paper sleep diary on behalf of their child to include bedtime (time child got into bed), time lights turned off, whether the event marker used to indicate bedtime and wake time was pressed at the correct time in the evening and morning, estimated time taken to fall asleep, wake up time in the morning and time got out of bed. Other important data for actigraphy data cleaning were collected, to include the timings and nature of any sedentary periods of activity after 6 pm, timings of any daytime naps, and the timings of periods when the Actiwatch was removed.

Data were cleaned to ensure that artefacts were removed and that the start of the intentional rest interval- bedtime was identified using a combination of the event marker, sleep diary, and the automatically calculated rest interval. This avoided relying solely on the software automatically calculated sleep intervals, which have poorer concordance with polysomnography (43, 44). Parental reporting of early morning final wake time may be inaccurate; therefore, sleep offset used the end of the autoscored rest interval. Intervals were extended to capture the entire sleep period if an additional 20 min after the end of the autoscored rest period but before the sleep diary indicated wake up time were coded as sleep by the software. Inter-rater reliability for 20% of the lights out time data in the AS group was excellent (45): intra-class coefficient: 97 (CI: 94–99).

### Analysis

Analyses were conducted using SPSS version 25. As some data were non-normally distributed, Mann–Whitney U and Wilcoxon Signed Ranks tests were used to compare actigraphy and questionnaire data between the AS and TD groups. Chi-squared analyses were used to compare categorical outcomes between groups. Linear regressions were used to explore the association between age and total sleep time and lights out time in both groups. To assess the degree of inter-individual variability in sleep, the SD of the total sleep time/wake after sleep onset of the group was divided by the group mean of the sleep parameter. Intra-individual variability was calculated by dividing the child’s SD in total sleep time/wake after sleep onset from the assessment period by the mean of the child’s sleep parameter from the assessment period. Effect size was calculated using Cohen R = Z÷√*N*.

## Results

### Describing Sleep Quality and Quantity

The results shown in **Table 2** demonstrate that children with AS had significantly earlier lights out times and poorer sleep efficiency than TD children. Differences in WASO also demonstrated a trend towards greater WASO in the AS group compared to the TD group, but the difference between the two groups was not statistically significant. No difference in sleep quality actigraphy parameters were found between children with AS who did or did not take sleep medication (see **Table S2**). When comparing the difference in actigraphy parameters between children in the AS group who did not receive melatonin (n = 9) and the TD group, there were no significant differences on any of the actigraphy parameters (p > .05), except earlier average lights out time for the AS group (AS group median 20:01, interquartile range 19:25–20:15, U = 35.0, p = .008). The same pattern of differences was found between children with AS who did receive melatonin (n = 11) and children in the TD group (average lights out time AS group median 20:04, interquartile range 19:32–20:33, U = 55.5, p = .023). No difference in the ratio of weekday versus weekend nights was found between the two groups. Furthermore, no difference between the average of total sleep time on weekday nights and weekend nights was found for children with AS (Z = -.859, p = .391) and TD children (Z = -.224, p = .823). Seven children with AS (age range: 4.0–13.21 years) had a nap on at least one of the days during the assessment period compared with three TD children (age range: 5.63–15.75 years). In the AS group, individual nap periods ranged from 5 to 240 min. In the TD group, individual nap periods ranged from 20 to 100 min.

**Table 2:** Grand median and interquartile ranges of actigraphy sleep and daily activity parameters across the assessment period and average duration of daytime naps in children with AS and TD children.

	AS	TD	Between-group comparisons		
			Mann–Whitney U/*X^2^*	*P*	Cohen’s R
Nights of actigraphy	7.0	7.0	169.5	.386	.14
Median (IQR)	(6.0–8.0)	(6.0–7.0)			
Ratio weekday/weekend nights	.27	.29	155.5	.210	.20
Median (IQR)	(.25–.29)	(.25–.32)			
Lights out time h:min	20:02	20:52	90.50	.003*	.47
Median (IQR)	(19:30–20:19)	(20:25–21:37)			
Sleep offset h:min	7:03	7:01	173.50	.473	.11
Median (IQR)	(5:58–7:41)	(6:29–7:23)			
Sleep onset latency min	19.32	15.42	197.50	.946	.01
Median (IQR)	(7.12–35.72)	(10.45–27.78)			
Wake After Sleep Onset min	76.39	56.67	129.0	.055	.30
Median (IQR)	(42.44–122.65)	(45.52–61.78)			
Sleep efficiency (%)	77.86	83.81	124.0	.040*	.33
Median (IQR)	(71.95–85.69)	(81.67–85.67)			
Total sleep time min	480.0	497.5	183.5	.655	.07
Median (IQR)	(459.83–548.0)	(479.5–525.88)			
Total sleep time weeknight mins	472.10	500.69	177.0	.534	.08
Median (IQR)	(446.34–538.06)	(475.90–528.43)			
Total sleep time weekend min	491.0	497.25	180.5	.598	.10
Median (IQR)	(459.56–572.56)	(479.94–542.13)			
Average duration of diurnal nap across assessment period for children who napped min Mean (SD)	31.08 (44.73)	7.86 (5.58)	–	–	–
Average timing of diurnal nap h:min Mean (SD)	15:59 (2:08)	16:18 (4:06)	–	–	–

*p < .05. Average duration of diurnal nap = total nap duration across assessment period according to sleep diary divided by number of nights of actigraphy data

According to the minimum recommended sleep time guidelines (46) seven children with AS and one TD child met the recommendations for their age group (*X^2^* = 5.63, 1, p = .018). See **Table 3** for distributions by age group.

**Table 3:** Proportions of children with AS and TD children meeting recommended minimum total sleep.

	AS	TD
	n (%)	n (%)
Children aged 4–5 years average TST ≥ 10 h	2 (40)	0
Children aged 6–12 years average TST ≥ 9 h	1 (10)	1 (9)
Children aged 13–15 years average TST ≥ 8 h	4 (80)	0

### Inter and Intra-Individual Variability in Total Sleep Time and Night Waking

Children with AS had greater variation in night to night (intra-individual variation) total sleep time and wake after sleep onset compared to TD children. Children with AS also had greater variation between individuals (inter-individual variation) in total sleep time and wake after sleep onset compared to TD children (see **Table 4**).

**Table 4:** Coefficient of variance statistics for total sleep time and wake after sleep onset between children and within an individual child’s assessment period in children with AS and TD children.

	AS	TD
Inter-individual coefficient of variance TST (%)	12	8
Inter-individual coefficient of variance WASO (%)	60	24
Intra-individual coefficient of variance TST (%)	15	10
Intra-individual coefficient of variance WASO (%)	53	24

### Sleep Hygiene, Symptoms of Sleep Disordered Breathing, and Excessive Daytime Sleepiness

The proportion of children with excessive daytime sleepiness did not differ between the two groups AS (n = 2 10.5%) and TD (0%), *X*^2^ = 2.22, p = .136. Children with AS did not have lower scores on the FISH measure of sleep hygiene compared to TD children. Children with AS were more likely to snore more than half of the time compared to TD children, but no other differences in sleep disordered breathing symptoms were observed (see **Table 5**).

**Table 5:** Scores on questionnaire measures of sleep hygiene, likelihood of dozing during daytime activities, sleep-related breathing disorders, and sleep disturbance for TD and AS groups.

	AS n	AS	TD n	TD	U statistic/X^2^	P value
Median sleep hygiene score on FISH† (IQR)	18	50.50 (46.75–56.50)	19	51.0 (47.0–54.0)	157.0	.670
Median pediatric Epworth sleepiness scale score † (IQR)	19	1.0 (0–6.0)	20	2.0 (1.0–3.0)	175.0	.669
Number (%) of children with severe settling problems	18	2 (11.1)	20	0	2.35	.126
Number (%) of children with severe night waking problems	16	13 (81.3)	20	1 (5.0)	21.75	< .001
Number (%) of children with severe early morning waking problems	19	4 (21.1)	20	1 (5.0)	2.25	.134
Always snores	16	0 (0)^a^	20	0 (0)	–	–
Snores more than half the time	17	4 (23.5)	19	0 (0)	5.03	.025
Snores loudly	16	1 (6.7)^a^	20	0 (0)	1.37	.241
Has heavy or loud breathing	17	7 (43.8)^a^	20	3 (15.0)	3.66	.056
Has trouble breathing, or struggles to breathe	16	1 (6.7)^a^	20	0 (100)	1.37	.241

a One parent reported “don’t know”.

### Associations Between Age and Total Sleep Time and Lights Out Time for Children With Angelman Syndrome and Typically Developing Children

**Figure 1:**
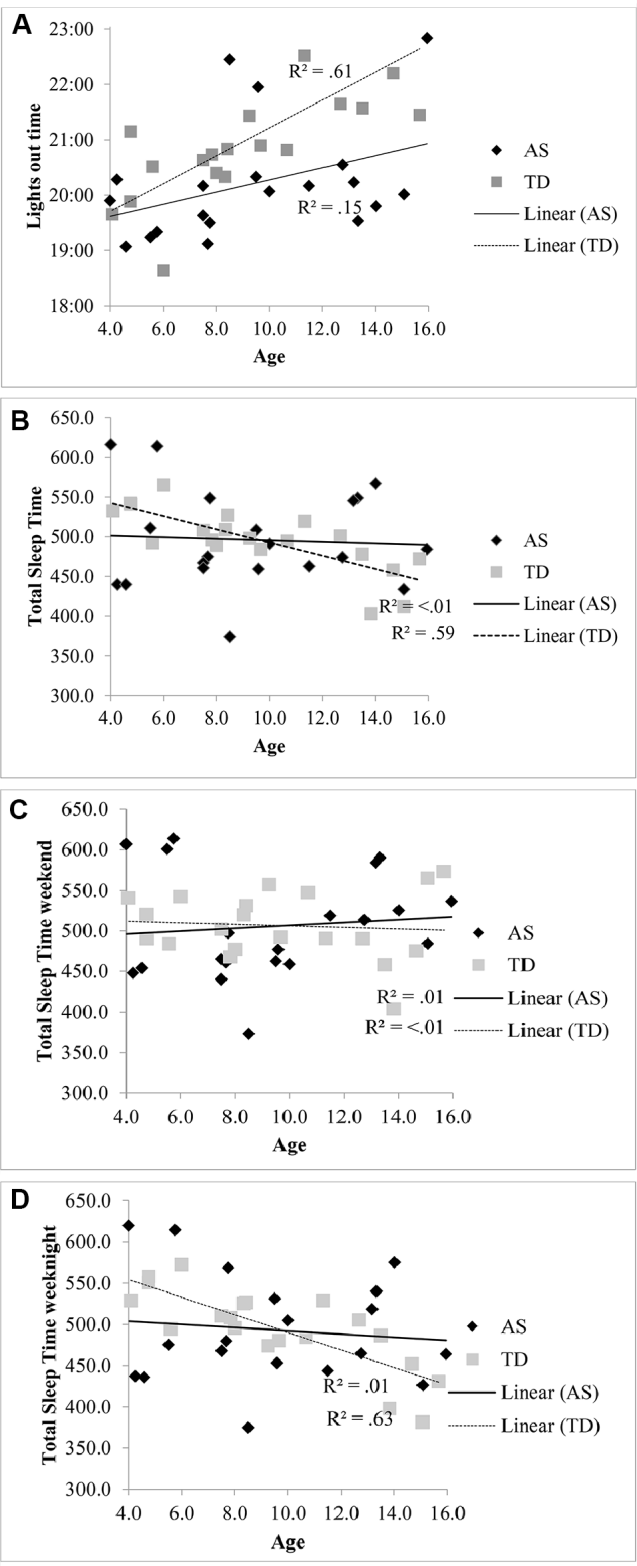
**(A)** Relationship between lights out time and age in children with AS and TD children. **(B)** Relationship between total sleep time and age in children with AS and TD children. **(C)** Relationship between total sleep time at the weekend and age in children with AS and TD children. **(D)** Relationship between total sleep time on weeknights and age in children with AS and TD children.

The lights out time for children with AS did not significantly vary with age [R^2^ = .15, F (1, 18) = 3.21, p = .09, β.39] compared to the association with later bedtimes for TD children with increasing age [R^2^ = .61, F (1, 18), 28.43 p < .001, β.78] (see **Figure 1A**). Total sleep in children with AS also did not significantly decrease with age [R^2^ < .01, F (1, 18) = .07, p = .799, β -.06] unlike the association observed in the TD group [R^2^ = .59, F (1, 18) = 25.57, p < .001, β -.77] (see **Figure 1B**). When the regressions were repeated separately for weekend averages (see **Figure 1C**) and weeknight averages (see **Figure 1D**), a decrease in total sleep time only associated with increasing age in on weeknights in the TD group [R^2^ = .63, F (1, 18) = 30.16, p < .001, β.79].

## Discussion

This is the largest actigraphy study of children with AS to date and the first to report wide inter and intra-individual variability in total sleep time and night waking in children with AS. As anticipated given the sample of children with AS with a parent reported sleep problem, 81% of children with AS had severe night waking problems according to a validated questionnaire. The data show that children with AS with parent-reported sleep problems had significantly earlier lights out times and poorer sleep efficiency than TD children and that a minority of children with AS napped during the day. No significant differences in total sleep time, wake after sleep onset and sleep offset time were found compared to TD children. Children with AS did not have an excess of daytime sleepiness compared to TD children, but were more likely to snore more than half the time (24 versus 0% of children) and, significantly, no difference in sleep hygiene practices were found between the two groups. Cross-sectional associations between lights out time, total sleep time and age indicated that increasing age is not associated with later bedtimes and decreased total sleep time in the AS group, unlike the relationship found for the TD group. The use of an age-matched TD contrast group in the first case–controlled actigraphy study of children with AS has enabled the benchmarking of impairment in sleep quality and differences in sleep timing.

These findings contrast from previous studies which indicate that individuals with AS have reduced total sleep time (see 19, 47). Our findings show that more children with AS sleep on average for the minimum recommended time than TD children. Given the inclusion criterion of children with AS with a parent-reported sleep disturbance, should the reported total sleep time be shorter in the wider AS population, we would have expected to have replicated this finding. It is possible that the TD group over-represented children with poor sleep quality, as in another study the average actigraphy assessed total sleep time in a large sample of children aged 9–11 years in the United Kingdom (48) met the minimum criteria advised by the American Academy of Sleep Medicine for this age group (45). The lack of relationship between age and bedtime in the AS group in contrast to the TD group is relevant to this finding. It has been suggested that it is environmental factors, as opposed to biological mechanisms, that may be responsible for the reduction in total sleep time with age in TD children (49, 50), as the decrease in total sleep time with age was only found on school days in one meta-analysis (47) and in the present study. This finding suggests that it may not be the case that the required sleep duration for TD children decreases, but that environmental constraints such as additional activities in the evening result in later bedtimes, as observed in **Figure 1A**, which reduces the time in bed and subsequent sleep period. Children with AS may not have the same demands on their time in the evening for extra-curricular and social activities, as their adaptive ability does not increase in line with their TD peers. Given the lack of difference in sleep onset latency between the groups, it would suggest that bedtimes in children with AS are aligned with their circadian phase. However, further research using melatonin assays to assess the timing of endogenous melatonin secretion onset is needed to explore whether children with AS have a circadian phase shift, as risk for circadian rhythm disorders has been identified in a small sample of individuals with AS (8). Further research is also needed both within TD and AS populations to investigate the hypothesised biological mechanisms of phase shift with age.

Despite the lack of difference between children with AS and TD children in total sleep time and wake after sleep onset, it is important to note that all parents of children with AS who participated in this study considered their child to have a sleep problem, and 81% had severe night waking problems according to a questionnaire definition. It is possible that heightened levels of night waking reported by parents in the AS group are due, in part, to greater self-soothing skills in the TD group, such that the TD children wake their parents less frequently and therefore night waking is under reported by parents of TD children. Additionally, it is possible that as children with AS are likely to have limited self-soothing skills due to their low adaptive ability, night wakings are extended as parents need to enter the child’s room which may be more stimulating and hinder the child’s ability to fall back to sleep quickly. Night waking duration varied substantially both between children with AS and from one night to another. This variability warrants further exploration. It is possible that the variance in night waking may be associated with nocturnal seizure frequency, however we do not have nightly reports on seizure frequency and duration to test this hypothesis. This has implications for assessment, namely that the severity of night waking using the average from children in one study cannot be generalised across children with AS. It is more important to assess sleep quality in children individually, and to evaluate night waking duration on multiple nights and consider the pattern of variation in sleep quality for each child. The detrimental impact of caring for a child with disturbed sleep cannot be underestimated, as parents of children with AS cite the impact of the impact of their child’s sleep problems on their own ability to function as a concern (51).

According to parental report of sleep disordered breathing symptoms, children with AS were more likely to snore more than half the time compared to TD children, although no child always snored. Preliminary evidence from a sleep questionnaire suggests that children with AS may be at risk of sleep disordered breathing compared to TD children (12), and habitual snoring reported for 24% of the AS sample is higher than the 9% reported for community samples using the same definition (52). However, these data were drawn from a small sample (n = 17), so caution is needed to generalise this finding to the wider AS population. However, given these rates of reported habitual snoring objective cardiorespiratory sleep studies should be performed with low threshold of clinical suspicion in this vulnerable group. Children with AS were not more likely to experience excessive daytime sleepiness according to the MESS. This finding does support previous literature demonstrating low prevalence of daytime sleepiness among individuals with AS (6, 13–15). The lack of excessive daytime sleepiness in both groups could reflect the lack of difference in average total sleep time between the groups. No differences in sleep hygiene practices were found between the two groups, therefore poorer sleep efficiency cannot be attributed to a distracting or uncomfortable bedroom environment or routines, particularly with regard to providing children with attention during the night. This is encouraging as children with AS are effective in obtaining social attention from adults (53). This suggests that whilst sleep hygiene needs to be examined on a case by case basis for each family, interventions for sleep in children with AS may not need to focus particularly on parent-child interactions at night, despite the strong motivation for social interaction observed in children with AS. However, it should be acknowledged that parents of children with AS may have had a different interpretation of brief interactions. Therefore, the role of parent-child interactions during night wakings needs to be explored using video footage and objective coding of interaction length and social approaches.

### Limitations

There are some limitations to the design of this study, predominantly the wrist placement of the Actiwatch for TD children and ankle placement for the majority of children with AS. A review suggests that whilst further research is needed, data obtained *via* ankle placement do not differ to those obtained *via* wrist placement (54). Due to children with AS’s limited receptive language, it was difficult to encourage children to wear the Actiwatch on their wrist and explain the value of wearing the watch. The Actiwatch was more often tolerated on the ankle when it was out of sight. On balance, it was felt that the statistical power obtained by including a greater number of children with a rare genetic syndrome in the study outweighed the risk of extraneous variance between the groups due to differential placement of the Actiwatch, however, it is possible that ankle placement may have underestimated wake after sleep onset if children had more limited leg movements compared to arm movements during the night. Whilst still the largest actigraphy sample of children with AS to date, the small sample size does limit the generalisation of these findings to other children with the deletion subtype of AS. Whilst polysomnography is often considered the ‘gold standard’ method of objectively assessing sleep, children with AS may struggle to tolerate wearing the equipment. Additionally, polysomnography can fail to capture habitual sleep/wake patterns in an ecologically valid sleep environment. Therefore, actigraphy was used to assess sleep in this population. Of note is that whilst we did not find differences in actigraphy parameters between children who were and were not administered medication to aid sleep, the cause of sleep disturbance in these two subgroups may be different.

Whilst it is informative to compare the cross-sectional relationships between sleep quantity and timing and age between children with AS and TD children, these data do not account for individual differences in children’s trajectories over time. Given the wide inter and intra-individual variation in total sleep time, it is important to approach these data with caution. A longitudinal study is required to confirm the difference in sleep quantity and timing with age in children with AS when interpersonal variation is accounted for. A longitudinal study could also identify the age at which sleep quality in children with AS diverges from the trajectory seen in TD children. We were unable to measure daytime melatonin in children with AS as the actigraphy and sleep diary data collection were facilitated by parents at home to maximise participation and also to ensure that data were ecologically valid. This does limit the discussion of the mechanisms that underpin interpersonal variation in sleep duration and quality among children with AS, as melatonin profiles in children with AS are disturbed (9). The measurement of endogenous melatonin should be a priority for future studies.

### Conclusions

This study generated the largest objective dataset of sleep quality parameters in children with AS. Sleep quality was characterised by a high degree of variability, both among children with AS with parent-reported sleep disturbance, and between nights for each individual child. This variability needs to inform further assessments of and interventions for sleep in children with AS, as averages of total sleep, even across a seven night period do not capture the difficulties with night waking highlighted by parental questionnaire report. No change in total sleep time with age unlike that found in TD children on weekday nights was hypothesised to be accounted for by the lack of environmental and social constraints on children with AS.

## Data Availability Statement

The datasets generated for this study will not be made publicly available due to small numbers of participants with a rare syndrome. Data may be identifiable.

## Ethics Statement

The studies involving human participants were reviewed and approved by University of Birmingham. Written informed consent to participate in this study was provided by the participants’ legal guardian/next of kin.

## Author Contributions

JT contributed to the design of the study, data collection, data analysis, and manuscript writing. CO contributed to the design of the study, supervision of the project, and manuscript editing. MH contributed to the design of the study and manuscript editing. HD contributed to data collection, data cleaning, and manuscript editing. AS contributed to data collection and manuscript editing. EC contributed to data collection and manuscript editing. PG contributed to the design of the study and manuscript editing. CR contributed to the study design, supervision of the project, and manuscript editing.

## Funding

This study was funded by Cerebra.

## Conflict of Interest

The authors declare that the research was conducted in the absence of any commercial or financial relationships that could be construed as a potential conflict of interest.

## Footnotes

Meltzer et al. (42) used 1 min epochs in comparison with polysomnography.
