# Supplementary material for: Multi-Method Assessment of Sleep in Children With Angelman Syndrome: A Case–Controlled Study
Source: Front Psychiatry. 2019 Nov 29;10:874. doi: 10.3389/fpsyt.2019.00874 (PMC6895248; doi:10.3389/fpsyt.2019.00874)
Supplement: Supplementary file 1 [file Table_1.docx]

**Table S1**: Medications used by children with AS

|  | Anti-epilepsy medication | Medication to aid sleep |  |
| --- | --- | --- | --- |
| #1 | Sodium Valporate 12ml, Clobazam 10mg | Melatonin 10mg |  |
| #2 | Kappra 11ml | Melatonin 5ml Alimenzino 75ml | Ranitidine 6.7m, Piriton 5ml |
| #3 | Kepra, Epilim and Clobozam |  |  |
| #4 | Kepra - 3ml twice per day and a Ketogenic diet |  |  |
| #5 | Sodium valproate 8oomg twice daily + 10mg Clobazam once daily |  | Lactulose 8mls twice daily, ibuprofen +paracetamol |
| #6 | Epilim 5ml BiD, Zarontin 4ml BiD | 4mg - Melatonin | Omaprazole (LOSEC) 20mg |
| #7 | Epilim 10mls bd. Ethosoximide 5mls (am) 6mls (pm) |  | Just finished antibiotics |
| #8 | Clobazam 10mg, Epilim 600mg x2 daily | Melatonin 10mg | Oreprazole 10mg x2 daily, 1 a x ido sachet |
| #9 | Clobazam 5mg x twice a day |  |  |
| #10 | Epilim 20ml Daily | Melatonin 10ml |  |
| #11 | Keppra (Levetiracetam) | Melatonin |  |
| #12 | Keppra twice a day/ sodium valporate 12.5ml twice a day | Melatonin 3mg once a day | Hyoscine 1mg/72 hours (1/2 patch every 3 days) |
| #13 | Epilim (valporate) 600mg am + 700mg pm, Clobazam 5mg bg | Melatonin 0.5mg (1/4 tablet) ON | Cetirizine 10mg on, Movicol paediatric 2 sachets on |
| #14 | Clobazam 5mgBD, Epilim 240mg BD | SR melatonin 4mg | Lansoprazole 15mg BD, montelokowst 5mg, magnesium supplement - well kid 10mls |
| #15 | Epilim 8ml x2 day |  |  |
| #16 | Clobazan, Eplim, Kepra | Alamemazine |  |
| #17 | Epilim 400mg x2 | Melatonin (Kidmel) 3mg |  |
| #18 | Epilim - 5ml am, 7.5ml pm. Clobazam 5mg pm |  | Movicol for constipation. Omeprozole - for 6 weeks follwoing infected gastostomy. Dacktacort for thrush |
| #19 | Sodium Valporate 280mg x2 daily | Melatonin 3ml daily | Buccal Midazolam, as needed (not given for ~ 36 months) |
| #20 | Lamotrigine 100mg BD, Clobazam 15mg BD |  | Nifedipine 10mg daily (5mg BD) |

**Table S2:** Sleep quality and timing in children with AS who were taking medication to aid sleep, compared to children who did not

|  | Taking sleep medication | Not taking sleep medication | Mann-Whitney U | P value |
| --- | --- | --- | --- | --- |
| Lights out time in hrs:mins  Median *(IQR)* | 20:04  (19:23-20:26) | 20:01  (19:30-20:17) | 43.0 | .843 |
| Sleep offset in hrs:mins  Median *(IQR)* | 7:01  (5:39-7:35) | 7:01  (5:39-7:35) | 38.50 | .579 |
| Sleep onset latency in mins  Median (*IQR)* | 21.21  (5.27-32.50) | 15.38  (9.13-39.94) | 42.0 | .782 |
| Wake After Sleep Onset in mins  Median *(IQR)* | 74.92  (23.05-133.44) | 77.86  (51.81-105.0) | 43.0 | .843 |
| Sleep efficiency in %  *Median (IQR)* | 74.37  (70.34-88.52) | 78.28  (76.55-86.11) | 37.0 | .501 |
| Total sleep time in mins  Median *(IQR)* | 475.0  (462.0-510.0) | 545.0  (433.81-615.0) | 42.50 | .812 |
